# Direct identification and molecular characterization of zoonotic hazards in raw milk by metagenomics using *Brucella* as a model pathogen

**DOI:** 10.1099/mgen.0.000552

**Published:** 2021-05-04

**Authors:** Josephine Grützke, Mayada Gwida, Carlus Deneke, Holger Brendebach, Michaela Projahn, Alexander Schattschneider, Dirk Hofreuter, Maged El-Ashker, Burkhard Malorny, Sascha Al Dahouk

**Affiliations:** ^1^​ Department of Biological Safety, German Federal Institute for Risk Assessment, Berlin, Germany; ^2^​ Department of Hygiene and Zoonoses, Faculty of Veterinary Medicine, Mansoura University, Mansoura, Egypt; ^3^​ Department of Internal Medicine and Infectious Diseases, Faculty of Veterinary Medicine, Mansoura University, Mansoura, Egypt; ^4^​ Department of Internal Medicine III, RWTH Aachen University Hospital, Aachen, Germany

**Keywords:** *Brucella*, detection limits, host depletion, metagenomics, raw milk

## Abstract

Metagenomics is a valuable diagnostic tool for enhancing microbial food safety because (i) it enables the untargeted detection of pathogens, (ii) it is fast since primary isolation of micro-organisms is not required, and (iii) it has high discriminatory power allowing for a detailed molecular characterization of pathogens. For shotgun metagenomics, total nucleic acids (NAs) are isolated from complex samples such as foodstuff. Along with microbial NAs, high amounts of matrix NAs are extracted that might outcompete microbial NAs during next-generation sequencing and compromise sensitivity for the detection of low abundance micro-organisms. Sensitive laboratory methods are indispensable for detecting highly pathogenic foodborne bacteria like *Brucella* spp., because a low infectious dose is sufficient to cause human disease through the consumption of contaminated dairy or meat products. In our study, we applied shotgun metagenomic sequencing for the identification and characterization of *Brucella* spp. in artificially and naturally contaminated raw milk from various ruminant species. With the depletion of eukaryotic cells prior to DNA extraction, *Brucella* was detectable at 10 bacterial cells ml^−1^, while at the same time microbiological culture and isolation of the fastidious bacteria commonly failed. Moreover, we were able to retrieve the genotype of a *Brucella* isolate from a metagenomic dataset, indicating the potential of metagenomics for outbreak investigations using SNPs and core-genome multilocus sequence typing (cgMLST). To improve diagnostic applications, we developed a new bioinformatics approach for strain prediction based on SNPs to identify the correct species and define a certain strain with only low numbers of genus-specific reads per sample. This pipeline turned out to be more sensitive and specific than Mash Screen. In raw milk samples, we simultaneously detected numerous other zoonotic pathogens, antimicrobial resistance genes and virulence factors. Our study showed that metagenomics is a highly sensitive tool for biological risk assessment of foodstuffs, particularly when pathogen isolation is hazardous or challenging.

## Data Summary

Raw data generated by next-generation sequencing have been uploaded to the European Nucleotide Archive (ENA) under study accession number ERP121102 (https://www.ebi.ac.uk/ena/browser/view/PRJEB37772). The bioinformatics analysis pipeline developed in our study is publicly available at GitLab (https://gitlab.com/bfr_bioinformatics/refsnper).

**Impact Statement**

Foodborne infections pose a major threat to public health, with 600 million cases reported annually worldwide. The monitoring of food is vitally important to prevent transmission of pathogens to humans through food consumption. Currently, next-generation sequencing is most commonly used to generate genomic data of bacterial isolates from patients and foodstuffs, which can be compared to trace foodborne outbreaks. Culture-independent metagenomics can speed up results, because time-consuming bacterial isolation is not required. Instead, the entire DNA of a food sample is analysed, but the relatively small proportion of pathogen DNA as compared to food-matrix DNA represents a challenge. As a result, pathogens are not detected or only small parts of their genomes can be recovered from the food sample, which significantly reduces the diagnostic value of this untargeted approach. To improve diagnostic applications in food control, the background signal from the food matrix has to be reduced to increase the amount of DNA retrieved from the micro-organisms. In our study, the advantage of eukaryotic cell depletion in food microbiology was proven for highly pathogenic zoonotic *Brucella* spp. in raw milk, which enabled the detection and molecular characterization of the pathogen without prior isolation.

## Introduction

*Brucella* spp. are fastidious, slow-growing zoonotic bacteria, often transmitted from animals to humans through the consumption of raw animal products, predominantly unpasteurized milk and cheese [1]. Bacterial isolation from food products is challenging, because brucellae are frequently overgrown by other bacteria of the food microbiome using classical culture methods. In addition, infected animals often shed the pathogen in low concentrations [<10^3^ c.f.u. (ml milk)^−1^] [2] and the infectious dose for humans is also very low, ranging from 10 to 100 bacteria. Therefore, genus-specific quantitative PCR (qPCR) is considered an adequate alternative diagnostic method to reliably identify *Brucella* spp. in foodstuff [3], but it does not allow for a detailed characterization of the pathogen, which is essential for risk assessment.

In contrast, whole-genome sequencing (WGS) of bacterial isolates provides a high resolution for the characterization of micro-organisms. Applying metagenomics as a culture-independent method to food affected by microbial contamination reveals a large number of sequencing reads originating from matrix material, which poses a challenge for the detection of low-abundance pathogens [4]. Sequencing of the food matrix can be avoided by amplification and sequencing of the bacterial 16S rDNA. However, with the so-called metabarcoding approach, various bacterial species cannot be resolved, making this method unsuitable for the universal detection of pathogenic bacteria [5]. Furthermore, non-bacterial taxa are not recorded and a more detailed characterization of pathogens, including genotyping or verification of virulence factors and antibiotic resistance genes, is impossible. Compared to the WGS commonly used for pathogen surveillance, the application of whole-metagenome sequencing (WMS) in food safety is still in the fledging stage [6]. Most of the present studies struggle with an insufficient percentage of microbial sequence reads, which are necessary to describe the relevant properties of a pathogen [5, 7–10].

Consequently, a drastic increase of sequencing depth is needed to get genome coverage sufficient for the detection of low-abundance species. According to Ottesen *et al.*, a 250-fold increase in sequencing depth is necessary to reach a coverage of one for all genomes in the sample [9]. Several studies improved detection limits by enrichment culture before metagenomics [7, 9, 10]. Enrichment for at least 8 h decreased the limit of detection from 10 000 c.f.u. Shiga toxin-producing *Escherichia coli* in 100 g fresh spinach to 10 c.f.u. [7]. However, enrichment culture only works for bacterial pathogens with high replication rates, such as *E. coli *, that are not overgrown by the food microbiome. Viruses and parasites remain unnoticed, as well as bacteria that require other growth conditions. In general, enrichment culture may alter microbial composition [7, 10]. Therefore, protocols for the enrichment of microbial DNA have been established, e.g. by using CpG-methylation in eukaryotic DNA [11] or differential centrifugation [12]. An alternative method to reduce undesired matrix signals is the selective lysis of eukaryotic cells based on the different membrane properties of eukaryotic and prokaryotic cells. In various studies, the depletion of human DNA in clinical samples increased the sensitivity of metagenomic analysis for the detection of microbes [13–19]. Enrichment of bacterial DNA by depletion of eukaryotic cells only slightly modifies the microbial composition of a broad range of bacteria compared to conventionally extracted DNA [13, 16, 17].

The goal of our study was to apply metagenomic shotgun sequencing for direct detection and detailed characterization of zoonotic pathogens in food matrices, with *Brucella* spp. in raw milk as a model. We used both artificially contaminated raw cow’s milk, and milk from naturally infected sheep, goats, buffalos and cattle, to establish metagenomic analysis in food samples and to prove the concept, respectively.

## Methods

### Collection of raw milk samples

A total of 151 midstream milk samples (2 ml each) were collected from 100 dairy cattle, 25 buffalos, 13 goats and 13 sheep following aseptic and standardized milking procedures. The animals were reared by 20 rural farming communities in Meet El-Amel, Aga District in Dakahlia Governorate, Delta region, Egypt. The animals included in our study either suffered from health disorders, such as subclinical mastitis (*n*=33), clinical mastitis (*n*=27), reproductive disorders (*n*=7) and abortion (*n*=31), or were apparently healthy (*n*=50) and gave normal birth (*n*=3). All samples were kept in a cooler until transport to the laboratory within 1 h after sample collection.

### Inoculation of raw cow’s milk

Raw milk was sampled from dairy cows kept on the experimental farm of the German Federal Institute for Risk Assessment (BfR), officially free of bovine brucellosis, and was inoculated with *Brucella abortus* bv. 1 (strain 544, NCTC 10093) with exponentially increasing concentrations ranging from 10 to 10^7^ cells (ml milk)^−1^. The bacterial solutions prepared for inoculation experiments were grown on BBL *Brucella* agar with 5 % horse blood (Becton Dickinson) for 3 days at 37 °C, to determine the actual number of cells by counting c.f.u., following the general assumption that one bacterial cell equates to one c.f.u. Three independent experiments were performed, resulting in three biological replicates with three technical replicates each. The artificially contaminated milk samples were inactivated by adding 100 % (v/v) ethanol to obtain a final concentration of 75 % (v/v), followed by an incubation at room temperature for at least 15 min and were stored at −80 °C until further use.

### Isolation of *Brucella* from milk

qPCR-positive milk samples were subjected to culture for bacterial isolation. In brief, 1 ml raw milk was diluted 1 : 10 in *Brucella* selective broth (Oxoid) [20] and incubated in a 25 ml cell culture flask at 37 °C and 5 % CO_2_. Over 6 weeks, 1 µl each was plated weekly on *Brucella* agar (Becton Dickinson) and selective agar (Oxoid) [20]. Suspicious isolates were identified as *Brucella* spp. using MALDI-TOF MS [21] and further characterized with classical microbiological methods [22].

### DNA extraction

DNA was extracted from *Brucella* milk isolates and raw milk samples (500 µl) using the DNeasy mericon food kit (Qiagen) according to the manufacturer’s protocol for 200 mg starting material. For eukaryotic cell depletion, 1 ml raw milk was centrifuged at 16 000 *g* and the pellet was resuspended in 200 µl PBS. The HostZERO microbial DNA kit (Zymo) was used to deplete eukaryotic cells and to extract DNA according to the manufacturer’s instructions, with a 3 min bead-beating step in a precooled TissueLyser LT (Qiagen) at 50 Hz. DNA concentration and purity were determined using the Qubit dsDNA HS assay kit with a Qubit 2.0 fluorometer (Thermo Fisher Scientific) and a NanoDrop ND-1000 UV-Vis spectrophotometer (NanoDrop Technologies).

### Detection of *Brucella* using qPCR

The genus-specific marker sequences *bcsp31* and IS*711* found in all *Brucella* spp. [23, 24] were amplified in a total volume of 25 µl including 5 µl template DNA using the QuantiFast pathogen PCR +IC kit (Qiagen) according to the manufacturer’s instructions. All samples were analysed in triplicates using a CFX96 Touch real-time PCR detection system (Bio Rad). *C*_t_ values ≤40 in both qPCR assays were empirically considered as a *Brucella *-positive test result.

### Next-generation sequencing

DNA libraries of *Brucella* milk isolates were generated with the Nextera XT DNA library prep kit (Illumina), and DNA libraries of raw milk samples with the TruSeq Nano DNA library prep kit, both according to the manufacturer’s instructions. In case the DNA concentration of a sample was below 2 ng µl^–1^, a fixed volume of 50 µl instead of 100 ng was used as input material. DNA was sheared using the M220 focused-ultrasonicator (Covaris). Next-generation sequencing was performed with NextSeq 500 and MiSeq (Illumina) in paired-end mode with 2×151 cycles and 2×251 cycles, respectively.

### Bioinformatics analysis

#### Quality control and read extraction

Raw reads were trimmed using fastp version 0.20.0 [25] with a mean phred-score of 30. For taxonomic classification, Kraken2 version 2.0.8-beta [26] with the RefSeq95 database and KrakenUniq version 0.5.8 [27] with the RefSeq bacteria database (downloaded on December 2, 2019) were applied on trimmed reads. Read extraction and blast confirmation were executed as previously described [5].

#### Species and strain prediction

Species and/or strain prediction based on extracted reads were performed with Mash Screen version 2.2 [28] using a winner-takes-all strategy or with the RefSNPer pipeline version 1.0.0 (https://gitlab.com/bfr_bioinformatics/refsnper). In the latter case, extracted reads were mapped to a set of complete or draft genomes available in the RefSeq database using bowtie2 version 2.3.5 [29], followed by the calculation of the reference genome coverage and SNP calling with SAMtools version 1.10 [30] and BEDTools version 2.29.0 [31]. All complete genomes of the genus *Brucella* were used for species prediction, and all complete and draft genomes of *B. abortus* for strain prediction. Subsampling of reads was performed with seqtk version 1.2-r94 using different seeds for each replicate (https://github.com/lh3/seqtk). SNP analysis of publicly available *B. abortus* assemblies was carried out with parSNP version 1.2 [32].

#### Assembly and genotyping

Metagenomic assemblies were generated using megahit version 1.1.3 [33]. Shovill version 1.0.4 (https://github.com/tseemann/shovill) was used to assemble WGS data and SPAdes version 3.10.0 for the assembly of extracted reads from WMS data. The quality and completeness of the resulting assemblies were analysed with quast 4 version 4.6.1 [34]. The *Brucella melitensis* core-genome multilocus sequence typing (cgMLST) analysis was carried out with chewBBACA version 2.0.16 [35] using a scheme of 2704 target genes [36]. SNP calling with the complete reference genome and phylogenetic analysis were conducted using snippy version 4.4.3 (https://github.com/tseemann/snippy) with mincov=3. For pairwise comparison of isolates and WMS data, bowtie2 and BCFtools were used. The community analysis was performed with R package vegan.

#### Prediction of virulence factors and antimicrobial resistance (AMR) genes

Virulence factors and AMR genes were identified by srst2 version 0.2.0 [37] with default parameters for WGS data (minimum coverage=90 %, minimum depth=5) and more relaxed criteria for WMS data (minimum coverage=30 %, minimum depth=1) using the Virulence Factor Database (VFDB, set A_nt) as downloaded on March 22, 2018 and National Center for Biotechnology Information (NCBI) AMRfinder database version 2019-10-30.1, respectively.

#### Graphical representation

Plots were generated in R with ggplot2 version 3.2.1, pheatmap version 1.0.12, plotly version 4.9.1 and vegan version 2.5-6. For graphic representation of phylogenetic trees, iTOL [38] and grapetree [39] were used.

## Results

### Detection of *Brucella* spp. in raw milk using metagenomic sequencing

**Fig. 1.**
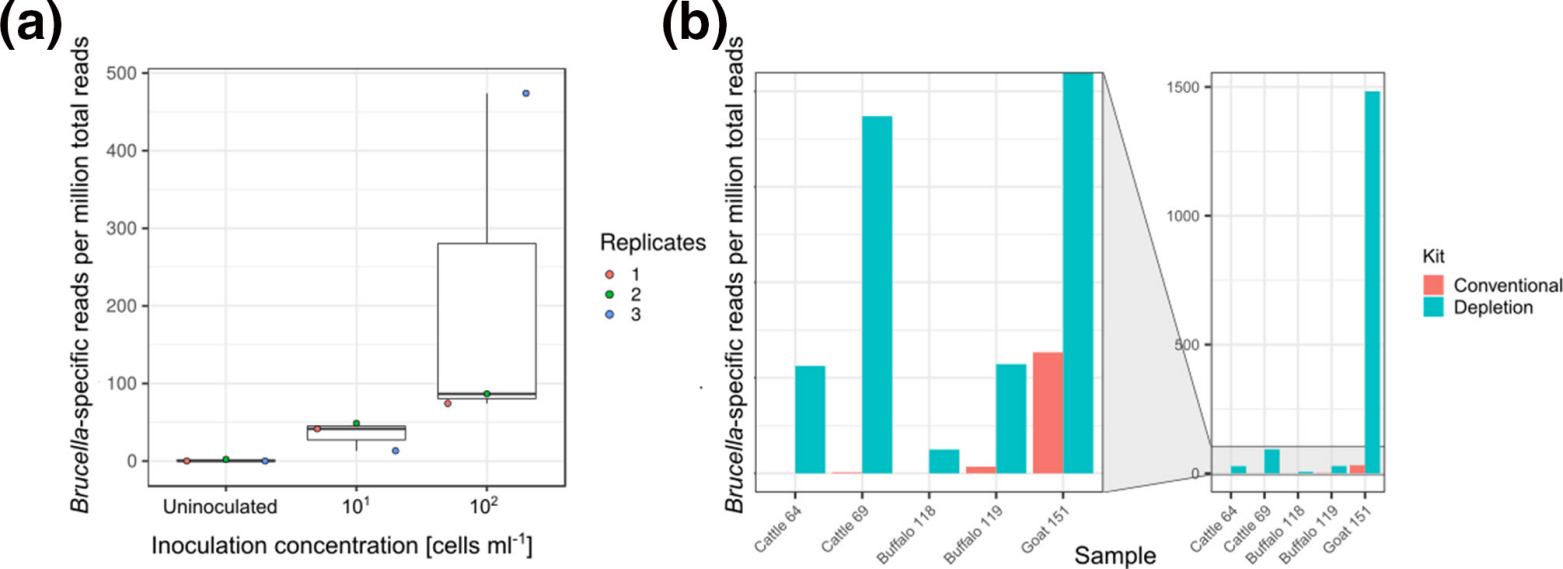
Eukaryotic cell depletion improves the detection of *Brucella* in raw milk by metagenomic analysis. (**a**) Detection of *Brucella *-specific reads per million total reads in artificially contaminated raw cow’s milk after eukaryotic cell depletion in three replicates. (**b**) *Brucella *-specific reads per million reads in milk samples from different dairy animals detected after conventional DNA extraction and DNA extraction with eukaryotic cell depletion.

We determined the molecular detection limits of two different genus-specific qPCRs targeting IS*711* and *bcsp31*. Independent of the target sequence, *Brucella* was reliably detected above 10^3^ bacterial cells (ml milk)^–1^ (Fig. S1, available in the online version of this article). Based on preliminary experiments (data not shown), the detection limit of metagenomics was tested at concentrations of 10^1^ and 10^2^ cells ml^−1^. Metagenomic sequencing of foodstuff generates high numbers of reads (>95 %) deriving from the food matrix, which results in a tremendous reduction of sensitivity for the detection of pathogenic micro-organisms. To enhance the fraction of bacterial sequences, we extracted DNA from inoculated and non-inoculated raw milk samples depleted of eukaryotic cells. In three independent sequencing experiments, we found 0.02–2.15 reads specific for *Brucella* per million reads in non-inoculated milk, 13–49 *Brucella* reads per million reads in milk samples inoculated at a concentration of 10^1^ cells ml^−1^ and 74–474 *Brucella* reads per million reads in milk samples inoculated at a concentration of 10^2^ cells ml^−1^ (Fig. 1a). Remarkably, with depletion of eukaryotic cells we were able to detect *Brucella* at very low concentrations between 22 and 31 bacterial cells (ml raw milk)^–1^, which corresponds to a 100-fold lower detection limit than the ones observed for qPCR assays testing the same samples.

In addition to artificially contaminated cow’s milk, we analysed raw milk samples from various ruminants potentially infected with *Brucella* spp. using qPCR and metagenomic sequencing. While 10 out of 151 raw milk samples were tested positive by the two genus-specific qPCR assays, *B. melitensis* was only isolated from a single goat's milk sample (no. 151). Three raw milk samples from buffalo (no. 118 and no. 119) and goat (no. 151) were selected on the basis of low *C*_t_ values in both qPCRs for metagenomic sequencing. Additionally, two samples with a *C*_t_ value >40 in the *bcsp31* qPCR were chosen from cattle (no. 64 and no. 69) (Table 1). The DNA of all samples was sequenced with and without previous eukaryotic cell depletion.

**Table 1.** Raw milk samples of potentially *Brucella *-infected animals selected for metagenomics

Sample no.	Origin	Health status	Mean *C*_t_ value for IS*711* (*n*=2)	*C*_t_ value for *bcsp31*
64	Cattle	Parturition	35.86	41.75
69	Cattle	Apparently healthy (q fever)	35.03	40.39
118	Buffalo	Abortion	35.25	39.07
119	Buffalo	Abortion	32.65	36.30
151	Goat	Apparently healthy	25.64	30.48

Using Kraken2 classification and blastn verification, we found 0–32 *Brucella *-specific reads per million reads with the conventional DNA extraction method and 6–1483 *Brucella *-specific reads per million reads with the eukaryotic cell depletion approach. Two (no. 64 and no. 118) out of five milk samples were considered *Brucella *-negative with 0 and 0.02 *Brucella *-specific reads per million reads, when conventional DNA extraction was applied. In contrast, all five samples were *Brucella *-positive when DNA extraction was conducted with eukaryotic cell depletion (Fig. 1b). Hence, *Brucella *-specific reads could be enriched 16- to 1400-fold by depleting eukaryotic cells.

In summary, metagenomic sequencing of total sample DNA after eukaryotic cell depletion led to an improved detection of *Brucella* in inoculated raw cow’s milk, with a 100-fold higher sensitivity than qPCR. Eukaryotic cell depletion also improved pathogen detection in naturally contaminated raw milk samples and revealed the presence of *Brucella* in two samples that were primarily considered as *Brucella *-negative, when conventional DNA extraction was applied.

### *Brucella* species and strain prediction from metagenomic datasets

**Fig. 2.**
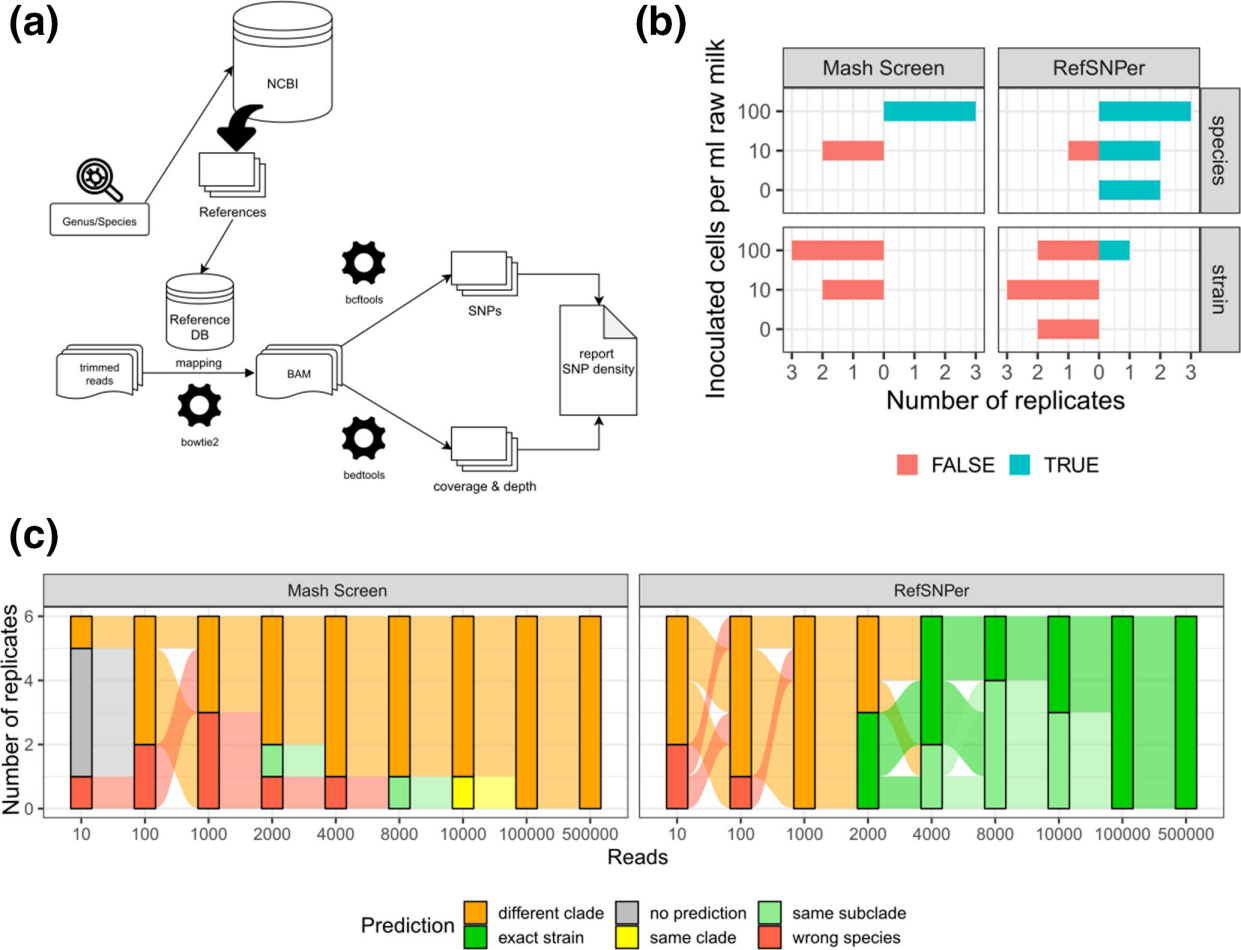
*
Brucella* species and strain prediction in metagenomic samples. (**a**) RefSNPer: a workflow for the identification of the closest reference strains. The input (a set of isolate or metagenomic sequences provided as paired fastq files) is mapped to a reference database. The reference database can be automatically generated for user-defined genera or species from draft and/or complete genome sequences available from the NCBI RefSeq database. Coverage, coverage depth (BEDTools) and number of putative SNPs (BCFtools) are determined for each reference genome. A summary file is generated that outputs the SNP density in each reference genome. (**b**) Species and strain prediction in raw milk inoculated with different numbers of *B. abortus* (strain 544) cells using Mash Screen and RefSNPer. Results of three replicates are given, excluding replicates without any prediction. (**c**) Species and strain prediction by Mash Screen and RefSNPer for an increasing number of *in silico* randomly subsampled reads from WGS data of *B. abortus* (strain 544) in six replicates.

Since qPCR does not allow for the differentiation of *Brucella* strains, we assessed whether metagenomic analysis will enable species and strain identification. We used Mash Screen and the newly developed bioinformatics pipeline RefSNPer for this purpose. RefSNPer executes read mapping to reference genomes followed by SNP calling and outputs the coverage, depth and number of SNPs for each reference genome (Fig. 2a). The objective behind this approach was to identify the most closely related genome exhibiting the smallest density of SNPs when compared to the sequences generated by next-generation sequencing (NGS).

First, we tested the ability of the two pipelines to predict the correct species and strain from the artificially contaminated raw milk samples, which were inoculated with *B. abortus* strain 544 (NCTC 10093). *Brucella *-specific reads classified by Kraken2 were extracted, analysed with Mash Screen and RefSNPer, and the correctness of predictions was evaluated (Fig. 2b, Table S1). With Mash Screen, we detected *B. abortus* in every replicate at a concentration of 100 bacterial cells (ml milk)^−1^. In milk samples inoculated at lower bacterial concentrations (10 cells ml^−1^), the correct species was only identified in one out of three replicates, and reported either not at all or incorrectly as *B. melitensis* in the other replicates. Strain prediction was always incorrect independent of bacterial concentrations. No results were obtained for the control samples, not inoculated with *Brucella *. In contrast, RefSNPer predicted the correct species in two out of three replicates at the lower concentration of 10 cells ml^−1^ and for all replicates at the higher concentration of 100 cells ml^−1^. The correct strain was predicted in one sample inoculated with the higher bacterial concentration.

Since unreliable strain prediction might be caused by a small number of reads, we tested the minimum number of reads needed for correct strain prediction using both bioinformatics tools. Increasing numbers of reads from WGS data of *B. abortus* strain 544 were randomly subsampled *in silico* in replicates (*n*=6) and analysed with both tools to define the phylogenetic distance to *B. abortus* strain 544 after core-genome SNP analysis with all publicly available complete and draft genomes of *B. abortus* (Figs 2c and S2, Table S1). We observed wrong species prediction with Mash Screen up to 4000 reads, whereas the correct species was already reliably predicted with 1000 subsampled reads using RefSNPer. Mash Screen analysis was not able to predict the correct strain, and resulted in the prediction of strains that locate to phylogenetically more distant clades (Fig. S2, Table S1). In contrast, RefSNPer predicted the expected strain or a very closely related strain in 50 % of the replicates using only 2000 subsampled reads for analysis and in all replicates using 4000 reads.

Mash Screen and RefSNPer analyses were also conducted on metagenomic data from naturally contaminated raw milk samples in order to determine the *Brucella* species after DNA extraction with and without previous eukaryotic cell depletion (Table 2). For most samples with less than 1000 *Brucella *-specific reads, a reliable prediction failed. Furthermore, the predictions could not be verified because we did not recover *Brucella* isolates from any of these samples. The result of species prediction changed from *B. abortus* to *B. melitensis* and from *B. abortus* to *Brucella ovis* in sample numbers 69 and 119, respectively, when a higher number of bacteria-specific reads was available due to eukaryotic cell depletion. While the identification of *B. melitensis* in cattle from Egypt is very likely, *B. ovis *, which is usually isolated from sheep, might be a false prediction for buffalo milk. Mash Screen analysis was not able to predict a species or most frequently *Brucella suis* was found in raw milk samples. Mash Screen did not predict the correct species from 2787 *Brucella *-specific reads in sample number 151 after conventional DNA extraction. In contrast, RefSNPer accurately identified the correct species as well as the closest complete reference genome *B. melitensis* strain 2008724259 (GCF_001715485.1) from the NCBI RefSeq database.

**Table 2.** *
Brucella* reads and species prediction from naturally contaminated raw milk samples

Sample no.	DNA extraction kit*	blast confirmed *Brucella *-specific reads	Predicted species	
			Mash Screen	RefSNPer
64	c	2	np	* B. abortus *
64	d	155	* B. suis *	* B. abortus *
69	c	24	np	* B. abortus *
69	d	810	* B. melitensis *	* B. melitensis *
118	c	0	np	np
118	d	56	np	* B. ceti *
119	c	50	* B. suis *	* B. abortus *
119	d	225	* B. suis *	* B. ovis *
151	c	2787	* B. suis *	* B. melitensis *
151	d	112 627	* B. melitensis *	* B. melitensis *

np, No prediction.

*c, Conventional DNA extraction; d, DNA extraction after eukaryotic cell depletion.

In summary, species and strain prediction were more reliable when RefSNPer was used. Mash Screen analysis never resulted in the expected strain. Using RefSNPer ≥1000 *Brucella *-specific reads were needed for reliable species prediction and ≥4000 reads for correct strain prediction.

### Comparison of genomic sequences from a *B. melitensis* isolate and metagenomic sequences from related goat's milk

To evaluate the potential of WMS for pathogen characterization in food matrices, we compared WGS data of three isolates from raw goat's milk with the *Brucella-*specific data from the WMS of the related sample (no. 151). We first conducted a RefSNPer analysis with the WGS reads and *Brucella *-specific reads extracted from WMS data, and consequently identified the genome sequence of *B. melitensis* strain 2008724259 (GCF_001715485.1) as the closest complete reference genome for comparison. The number of *Brucella *-specific sequencing reads was about 12-fold higher in the WGS than in the WMS dataset, also resulting in a higher coverage depth of 39- to 55-fold after WGS of isolates in comparison to 5.3-fold on average after WMS of the contaminated raw milk sample. WGS and WMS datasets covered the reference genome almost completely with 99.99 and 99.05 %, respectively. Pairwise comparison of the three isolate sequences resulted in 2–5 SNPs and pairwise comparison of the metagenome to the assemblies from WGS of the isolates resulted in 43–44 SNPs, but only 14–16 SNPs were supported by ≥3 reads as shown in a SNP matrix (Fig. S3).

**Fig. 3.**
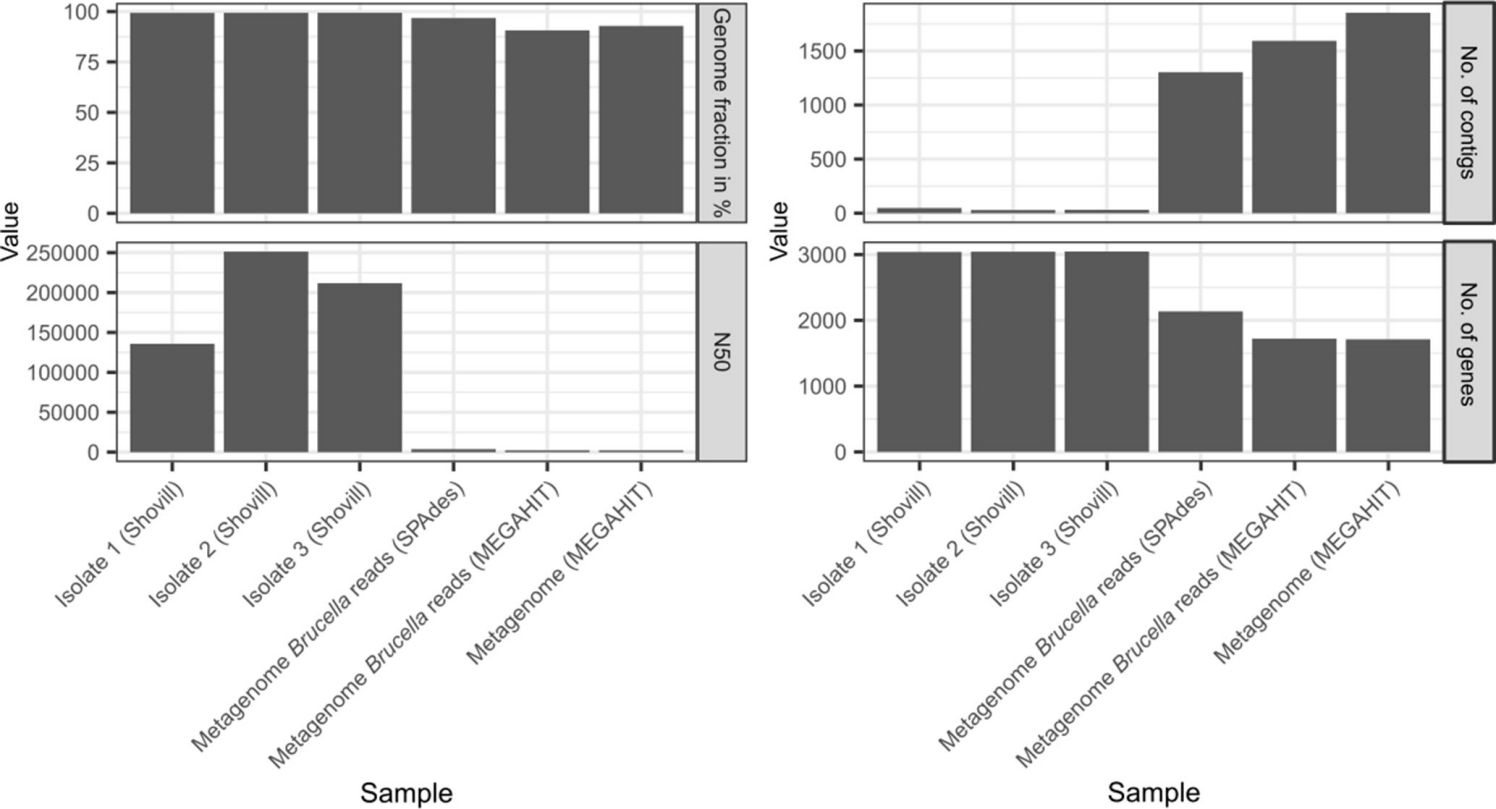
Statistics for different assembly strategies. Assemblies were generated from the whole metagenome (metagenome), from *Brucella *-specific reads extracted from the metagenome (metagenome *Brucella* reads), and from WGS data of isolates originating from the related raw milk sample. The genome fraction, N50, number of contigs and number of genes recovered from these assemblies were analysed using *B. melitensis* strain 2008724259 (GCF_001715485.1) for reference.

In a next step, we tested two different assembly strategies: assemblies generated from all metagenomic sequences (metagenome) and from *Brucella *-specific reads extracted from the metagenome (metagenome *Brucella* reads) with SPAdes and megahit. These assemblies were evaluated with *B. melitensis* strain 2008724259 (GCF_001715485.1) as a reference genome (Fig. 3). A metagenomic assembly of the WMS data resulted in 1857 contigs covering 92.9 % of the reference genome. *Brucella* read extraction and assembly resulted in fewer contigs (*n*_SPAdes_=1303, *n*_MEGAHIT_=1593) and a higher recovery of the reference genome (96.8 %) when SPAdes was used. When WGS data of isolates were assembled, 99.4 % of the genome could be recovered and the number of contigs varied between 29 and 48.

**Fig. 4.**
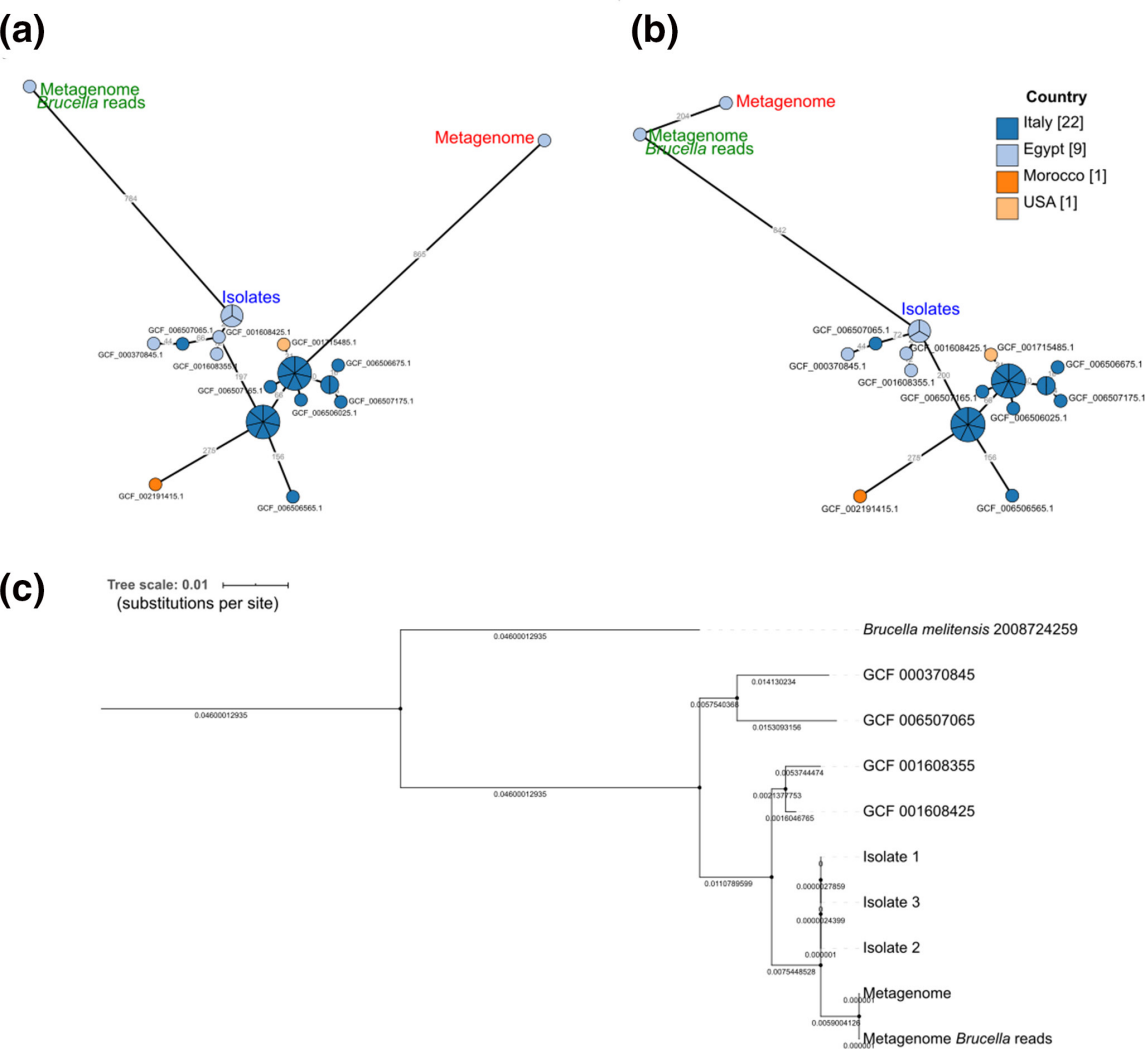
Genomic typing of *Brucella* from Egyptian raw milk samples by cgMLST (**a, b**) and SNP analysis (**c**) using WGS and WMS datasets. (**a**) Excerpt of a minimum spanning tree (Fig. S4 gives an overview) based on cgMLST allelic profiles of 336 publicly available genomes, and assemblies of WGS and WMS data from raw milk sample number 151. Assemblies from WMS data were generated either by metagenomic assembly (metagenome) or after extraction of *Brucella *-specific reads (metagenome *Brucella* reads). Numbers at the branches stand for allelic distances, and branches presenting less than 10 alleles difference are collapsed. Assemblies of WGS data of isolates and *Brucella *-specific reads were generated either with Shovill and SPAdes, respectively (**a**), or with megahit (**b**). (**c**) Phylogenetic relationship of four closely related publicly available draft genomes and WGS and WMS data obtained from raw milk sample number 151 based on core SNP distances illustrated in a maximum-likelihood tree.

A cgMLST analysis was conducted with all publicly available draft and complete *B. melitensis* genomes (*n*=336) retrieved from the RefSeq database, the assembled genome sequences of isolates, the *Brucella *-specific reads (extracted from metagenomes) assembled with SPAdes and the metagenomic assemblies of WMS data. The isolates and the assembly of *Brucella *-specific reads extracted from WMS data clustered in the same subclade of a minimum spanning tree based on cgMLST allelic profiles (Fig. S4), whereas the metagenomic assembly of WMS data was found in a more distant clade (Fig. 4a). The isolates analysed in our study and the assembled *Brucella *-specific reads from WMS data clustered more closely together with three other isolates from Egypt (GCF_000370845.1, GCF_001608425.1, GCF_001608355.1) and one isolate from Italy (GCF_006507065.1) than with the metagenomic assembly from WMS data. The phylogenetic distance of the metagenomic assembly resulted from the relative low number of matching alleles, with 1105 and 682 when isolate 2 was compared to the assembled *Brucella *-specific reads and the assembled total metagenomic dataset, respectively. To test the impact of the assembler chosen for analysis, we also assembled the WGS data of isolates and *Brucella *-specific reads (extracted from the metagenome) with megahit and conducted a cgMLST analysis. Consequently, the metagenomic assembly from WMS data now clustered together with isolates and assembled *Brucella *-specific reads (Fig. 4b). Hence, cgMLST results clearly depend on the assembly algorithm, which was also proven for the WGS data of isolates that clustered differently (Fig. 4a, b).

A SNP analysis using the closest complete reference genome available in NCBI, previously identified by RefSNPer, was performed. Besides sequencing data of sample number 151, including genomic sequences of isolates, WMS data and *Brucella *-specific reads extracted from WMS data, we analysed publicly available genomes (GCF_000370845, GCF_006507065, GCF_001608355 and GCF_001608425) found in the same cgMLST cluster. The *B. melitensis* isolates from goat's milk revealed 190 SNPs and the metagenomic data 179 SNPs in comparison with the reference genome. Relationships based on SNP analysis were depicted in a maximum-likelihood tree with *B. melitensis* strain 2008724259 (GCF_001715485.1) as an outgroup (Fig. 4c). The three isolates and the metagenomic samples shared the same SNPs at 179 positions and revealed a distance of 11 SNPs, while the publicly available genomes in the same cgMLST cluster showed a SNP distance of 21–72 to the isolates in our study. In contrast to cgMLST analysis, the relationship of the WGS data of isolates was much closer with the WMS data gained from our experiments than with the four publicly available genomes.

In summary, a close relationship between isolates and *Brucella* sequences from WMS data was proven, but they were not found in the same subclade using high-resolution SNP analysis. There was no difference between the total WMS dataset and the extracted *Brucella *-specific reads in the SNP analysis. In contrast, our cgMLST analysis was less robust and dependent on the assembler used.

### Milk microbiota and associated zoonotic bacterial pathogens

**Fig. 5.**
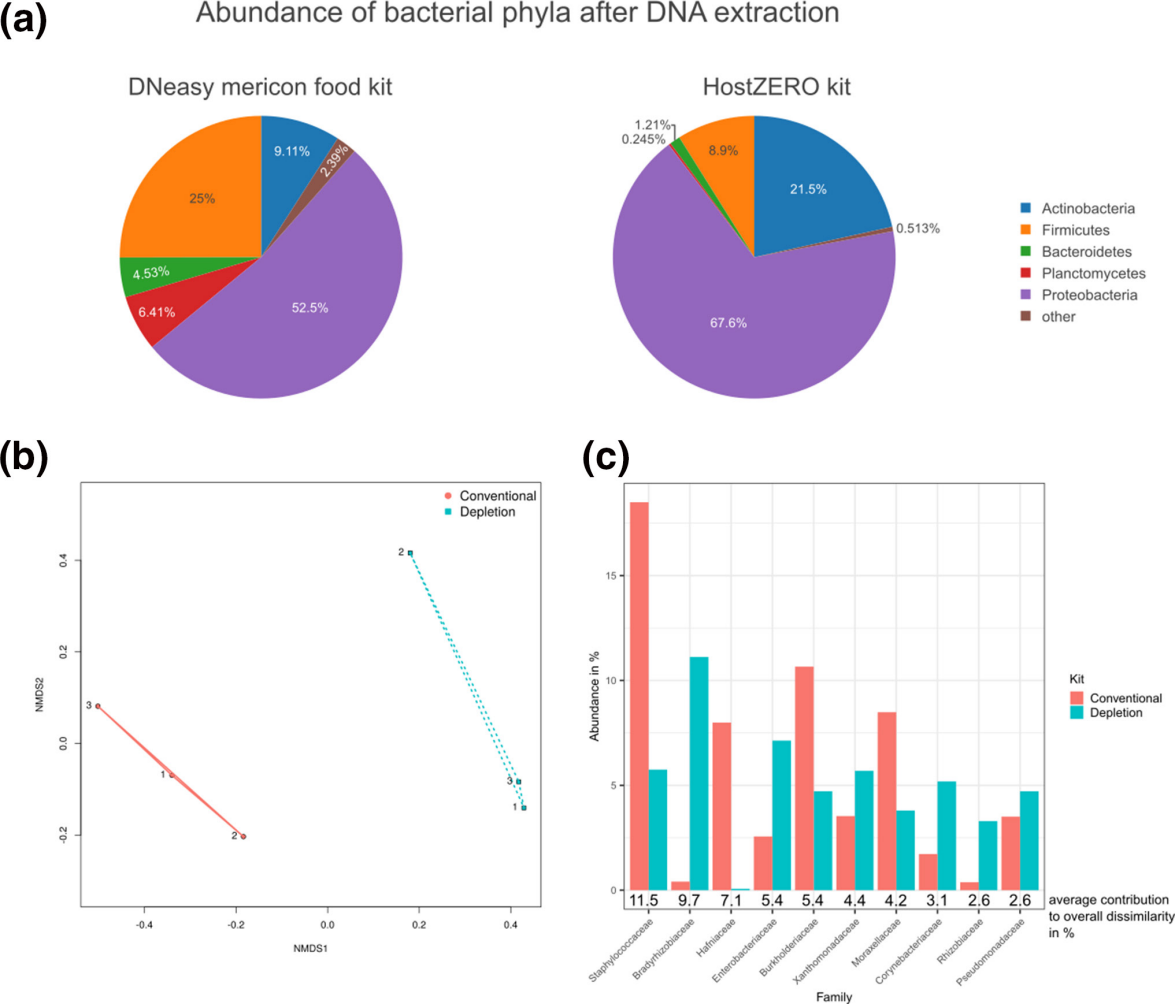
Analysis of the effects of different DNA extraction strategies on the abundance of bacterial phyla in pure cow’s milk. The prokaryotic composition of raw milk used for inoculation experiments was determined by KrakenUniq after conventional DNA extraction with the DNeasy mericon food kit, and after eukaryotic cell depletion and DNA extraction with the HostZERO kit. (**a**) Mean abundances (%) of the bacterial phyla most prevalent in three replicates are presented in a pie chart for each extraction kit. (**b**) Non-metric multidimensional scaling (NMDS) analysis based on Bray–Curtis dissimilarity of the detected bacterial families in all samples after conventional DNA extraction (red) and DNA extraction with eukaryotic cell depletion (blue). (**c**) Mean abundances (%) of the families that were most discriminating between the two DNA extraction kits obtained by analysis of similarity percentage (SIMPER) using Bray–Curtis dissimilarity are presented in a bar chart together with the mean contribution to the overall dissimilarity.

In addition to zoonotic pathogens, we investigated the composition of the prokaryotic community in raw milk in order to assess the effects of eukaryotic cell depletion. We first analysed the taxonomic composition of bacterial phyla in the pure raw milk used for inoculation experiments (Fig. 5a). Regardless of the extraction method applied, the major phylum was *Proteobacteria *. The fractions of *Firmicutes *, *Planctomycetes* and *Bacteroidetes* were larger in total DNA extracts than in DNA extracted after eukaryotic cell depletion, and vice versa for the fractions of *Proteobacteria* and *Actinobacteria *. The differences in the relative abundance of bacterial phyla in milk, depending on the DNA extraction method used, could be confirmed at family level by using a non-metric multidimensional scaling analysis (NMDS), which revealed two different clusters (Fig. 5b). While analysis of similarities (ANOSIM) showed a high within-group similarity and dissimilarity between groups (R=1), the difference between the groups was not statistically significant (*P*=0.1). The analysis of similarity percentages (SIMPER) showed that these dissimilarities were mainly caused by *Staphylococcaceae *, *Hafniaceae *, *Burkholderiaceae* and *Moraxellaceae *, which were more abundant when conventional DNA extraction was applied, and *Bradyrhizobiaceae *, *Enterobacteriaceae *, *Xanthomonadaceae *, *Corynebacteriaceae *, *Rhizobiaceae* and *Pseudomonadaceae *, which were more abundant after eukaryotic cell depletion (Fig. 5c).

**Fig. 6.**
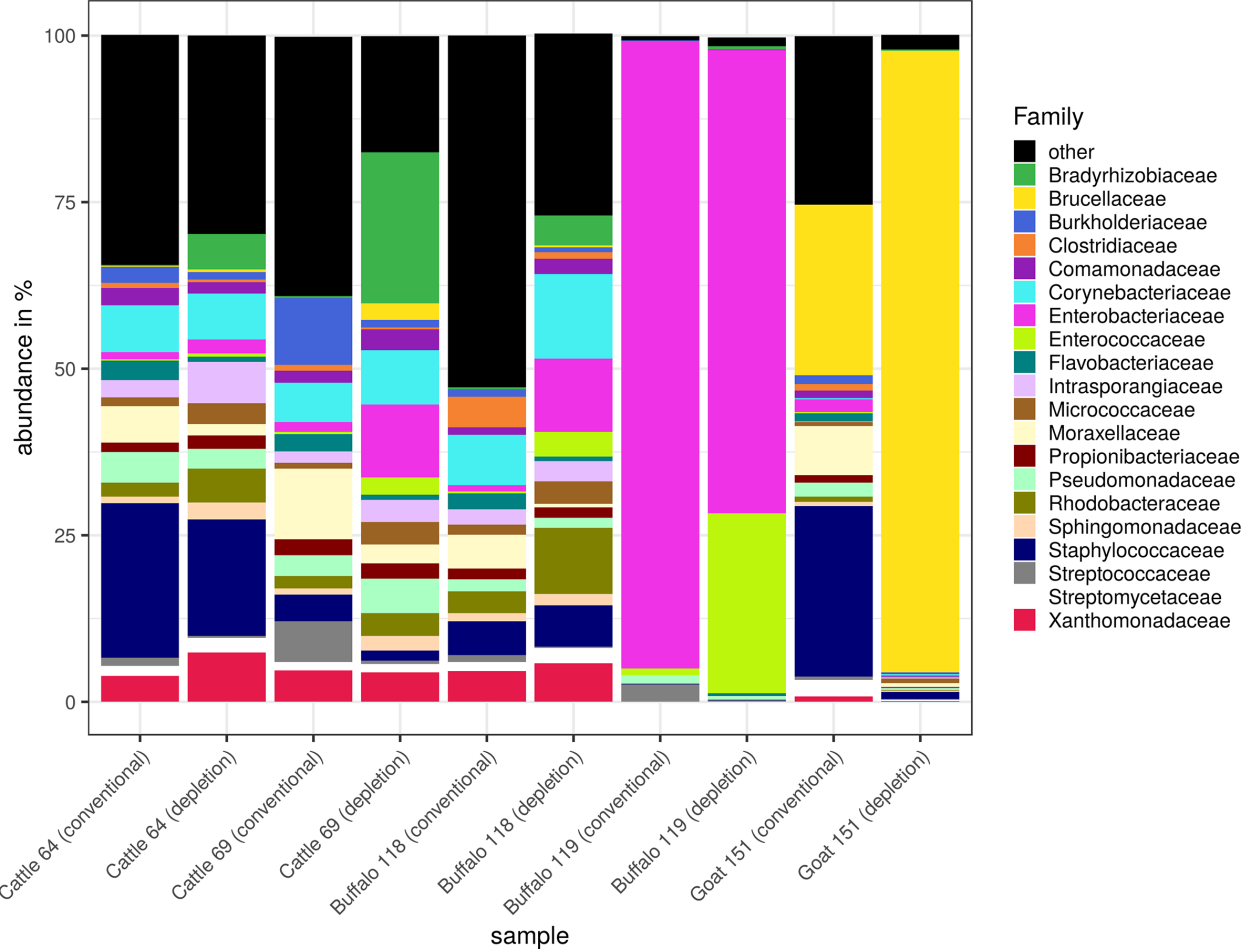
Taxonomic composition of raw milk samples from different dairy animals. The prokaryotic composition in raw milk after conventional DNA extraction with DNeasy mericon food kit and after eukaryotic cell depletion and DNA extraction with HostZERO kit was determined using KrakenUniq. Relative abundances (%) of bacterial families in different milk samples are presented as stacked bar graphs, with both DNA extraction kits next to each other for comparison.

We also analysed raw milk samples from different dairy animals for the most abundant bacterial families (Fig. 6). In general, the profiles found in sample numbers 64, 69 and 118 were quite similar. In these samples, the fractions of *Bradyrhizobiaceae *, *Enterobacteriaceae *, *Rhodobacteraceae* and *Micrococcaceae* were larger after DNA extraction with eukaryotic cell depletion than without, whereas the fractions of *Flavobacteriaceae* and *Moraxellaceae* were smaller. In sample number 119, the fraction of *Enterococcaceae* was strongly increased after depletion of eukaryotic cells, and the fraction of *Streptococcaceae* was slightly decreased. In general, we observed a smaller fraction of low abundance families after eukaryotic cell depletion together with decreased species richness (data not shown). In sample number 151, the fraction of *Staphylococcaceae* and *Moraxellaceae* was strongly decreased, while the fraction of *Brucellaceae* was enriched after eukaryotic cell depletion. Furthermore, we observed a high prevalence of *Enterobacteriaceae* in sample number 119, usually present in the gut of mammals and, therefore, indicating a faecal contamination of the milk.

**Fig. 7.**
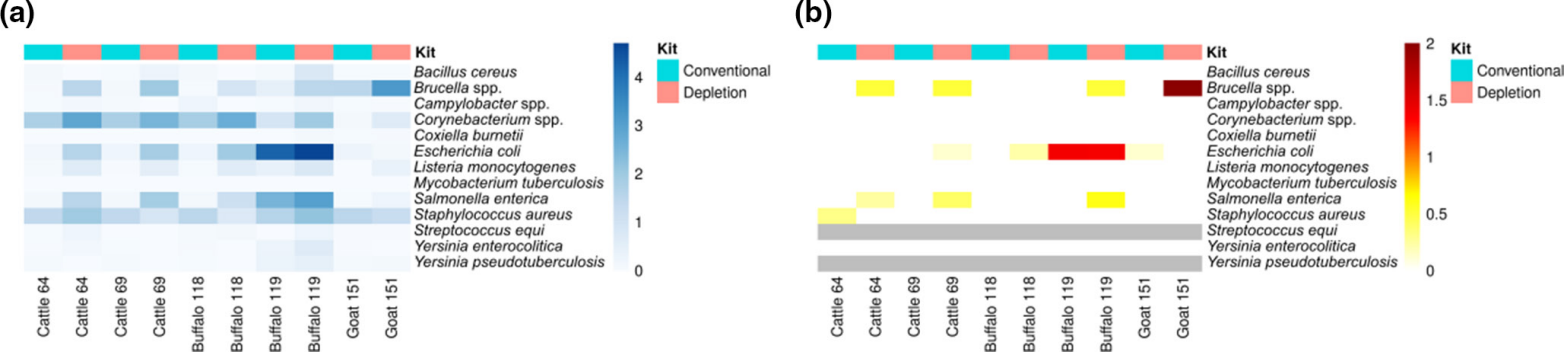
Hazardous bacterial species and their virulence factors in raw milk samples from different dairy animals. Detection of potentially hazardous bacterial species and their virulence factors in raw milk after conventional DNA extraction and after DNA extraction with eukaryotic cell depletion. (**a**) Classified species-specific reads per million total reads depicted in a heatmap. (**b**) Percentage of virulence factors detected in a species in relation to the total number of known virulence factors in the respective species according to VFDB was calculated. Grey colour indicates the absence of virulence factors in these species in the VFDB. To improve data presentation in the heatmaps, values were transformed by 1+log_10_.

Additionally, we looked for hazardous bacteria [40] in the milk samples from Egypt that can be transmitted through the consumption of raw milk and products thereof using KrakenUniq. Besides *Brucella* spp., other potentially pathogenic species such as *E. coli *, *Salmonella enterica* and *Staphylococcus aureus* were detected in almost every sample (Fig. 7a). A few samples contained *Corynebacterium* spp., *Yersinia* spp., *Listeria monocytogenes* and *Bacillus cereus* in low abundance. For many of the pathogenic species (namely *Bacillus cereus *, *Brucella* spp., *Corynebacterium* spp., *E. coli *, *Salmonella enterica *, *Staphylococcus aureus* and *Yersinia* spp.), we observed an improved detection when eukaryotic cell depletion was applied prior to DNA extraction, showing the applicability of the method not only to Gram-negative *Brucella* spp., but also to Gram-positive bacteria such as *Bacillus* and *Staphylococcus *.

Although we detected many potentially pathogenic bacterial species, we only identified virulence factors of *E. coli *, *Brucella* spp., *Salmonella enterica* and *Staphylococcus aureus* (Fig. 7b, Table S2). A reason for that might be the low genome coverage for most species. In sample number 119, *E. coli* was highly abundant, and was found together with typical indicators of faecal contamination such as *Enterococcus faecalis* and *Enterococcus faecium *. Seventy *E. coli *-specific virulence factors could be identified in this sample. No virulence genes typical for enteropathogenic *E. coli* encoding Shiga toxins, heat-stable or heat-labile enterotoxins, or other pathogenicity factors such as intimin (*eae*) and invasin plasmid antigen (*ipaH*), were detected. However, six type III secretion effectors were found, which are encoded on the pathogenicity island locus of enterocyte effacement (LEE), mediating the formation of attaching and effacing lesions in the intestinal epithelium.

In summary, there are differences in the composition of bacterial families depending on the DNA extraction kit applied. If eukaryotic cells were depleted, the detection of several pathogenic bacterial species could be improved.

### Detection of AMR genes in raw milk

**Fig. 8.**
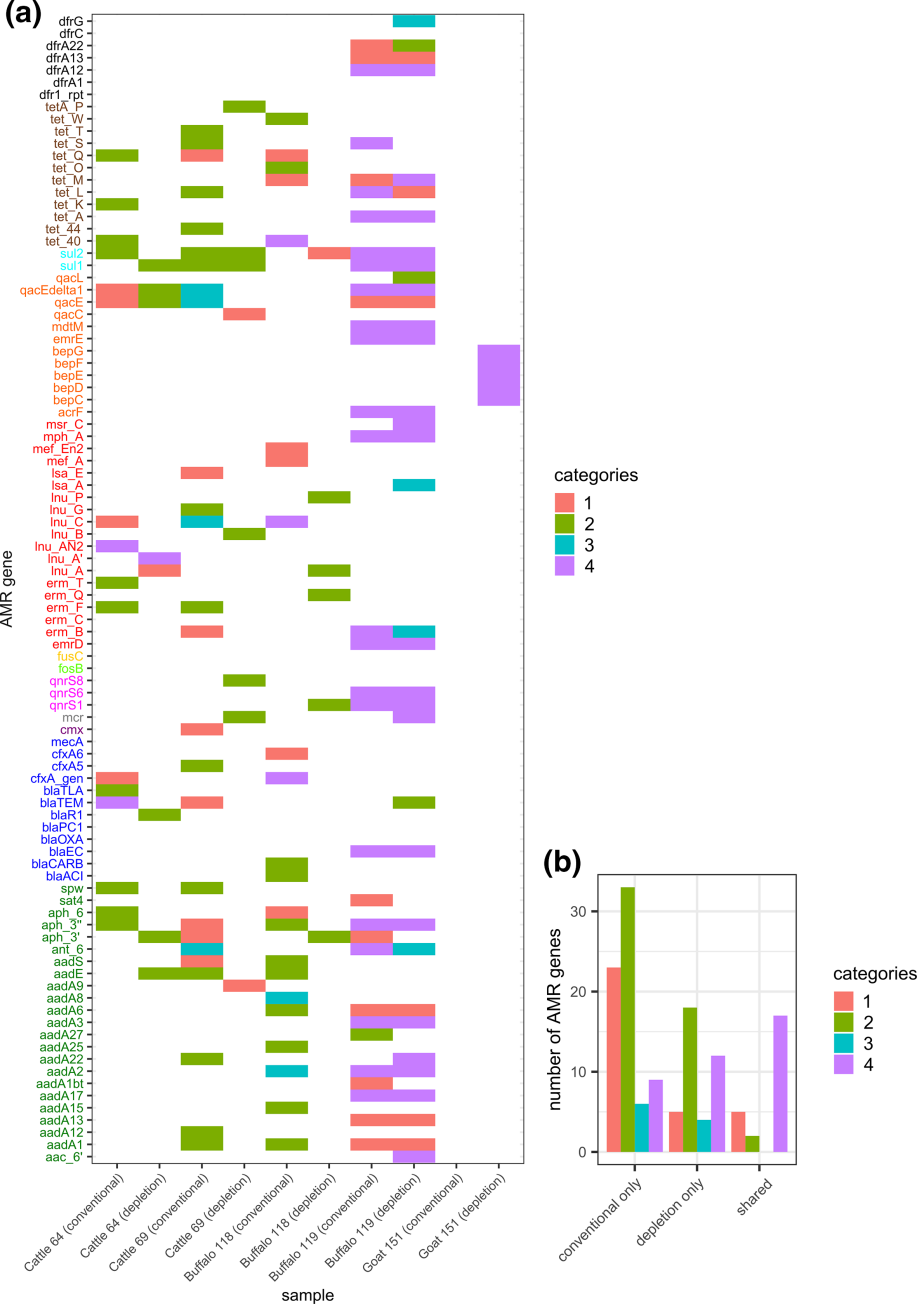
Presence of antibiotic resistance genes in raw milk. WMS data of raw milk samples were screened for AMR genes after conventional DNA extraction with a DNeasy mericon food kit or after eukaryotic cell depletion and DNA extraction with a HostZERO kit. The probability of gene presence was categorized into four classes according to coverage and depth: category 1, very low evidence – truncated gene (coverage <90, depth >1.5); category 2, low evidence – very low abundance (coverage <60, depth <1.5); category 3, medium evidence – low abundance (coverage >60, depth <1.5); category 4, high evidence (coverage >90). (**a**) Colours of gene names refer to AMR classes: aminoglycoside (green), β-lactam (dark blue), chloramphenicol (violet), colistin (grey), fluoroquinolone (pink), fosfomycin (light green), fusidic acid (yellow), macrolide (red), multidrug resistance (orange), sulfonamide (cyan), tetracycline (brown), trimethoprim (black). (**b**) Number of AMR genes exclusively detected after conventional DNA extraction (conventional only) and after eukaryotic cell depletion (depletion only) or by both DNA extraction methods (shared).

The screening of metagenomic datasets for AMR genes revealed 94 genes with a coverage >30 %, distributed across twelve different antimicrobial classes (Fig. 8a). Several of these genes confer resistance to last-resort antibiotics. The detected AMR genes were categorized by certainty of evidence considering gene coverage and coverage depth. In general, DNA extraction methods again affected results. The depletion of eukaryotic cells prior to DNA extraction by differential lysis resulted in lower retrieval rates of AMR genes (Fig. 8b), especially in case of a lower certainty of evidence. Across all samples, only 38 genes revealed high certainty (coverage >90). Seventeen out of these were detected independent of the DNA extraction method applied. The detection of 12 genes could be improved by eukaryotic cell depletion. The highest prevalence of AMR genes (*n*=17) belonging to category 4 [high evidence (coverage >90)] were found in sample number 119, which was from a buffalo. This sample also contained a high proportion of *Enterobacteriaceae* (>60 %) that might be the reservoir of verified AMR genes. Sample number 151, from a goat, contained several components of the multidrug efflux RND (Resistance–Nodulation–Division) transporter, which is assumed to confer resistance to various antibiotics such as tetracycline, doxycycline and fluoroquinolones [41]. These genes can be also assigned to the *Brucella* genome, and were found in the isolates obtained from the milk sample (data not shown).

## Discussion

Our study demonstrates that metagenomic sequencing can be used for direct pathogen detection and characterization in raw milk without pre-enrichment culture. For a proof of concept, we chose *Brucella* as the ideal model pathogen because it is naturally shed in milk of infected ruminants, is difficult to isolate from complex samples due to its fastidious growth, exhibits a high homology among its species and has a low infectious dose, which is why it is considered as highly pathogenic. In our study, we determined the detection limits of metagenomic sequencing and qPCR to correctly identify *Brucella* in artificially contaminated raw milk. Metagenomic sequencing of DNA after eukaryotic cell depletion decreased the detection limit for *Brucella* in raw milk to <20 c.f.u. ml^−1^. Hence, metagenomics showed a higher sensitivity than the gold standard in molecular diagnostics of brucellosis, which is a genus-specific qPCR after conventional DNA extraction (>1000 cells ml^−1^). Our results are comparable to previously reported detection limits of metagenomics for *E. coli* in spinach with 10 c.f.u. g^−1^ after 5 h of enrichment [7]. Eukaryotic cell depletion also performed quite well in naturally contaminated raw milk, although most brucellae are known to reside in milk macrophages [42]. Furthermore, ethanol pre-treatment of the milk to inactivate pathogens before DNA extraction did not impair the performance of eukaryotic cell depletion. In a recent metabarcoding study, different kits used for the depletion of host signals in infected human tissue samples were compared, and bacterial DNA could be enriched more than 10-fold by the HostZERO kit [19]. After eukaryotic cell depletion with the same method, we proved a similar enrichment of *Brucella *-specific reads in raw milk samples, which makes this approach suitable for pathogen detection in animal food products.

Since *Brucella* genomes display a strong sequence homology among species, a monophyletic genus has been assumed [43, 44]. The close relationship of *Brucella* spp. makes it challenging to determine the species when only a few sequence reads are available. In our study, we compared two different bioinformatics approaches for species and strain prediction: Mash Screen and RefSNPer. At low read numbers (<8000 reads) *Brucella* species prediction is much more reliable with RefSNPer than with Mash Screen. However, RefSNPer also needs more than 1000 reads to reliably identify the species. Mash Screen was not able to predict *Brucella* strains, whereas RefSNPer enabled strain prediction but at least 2000 genus-specific reads were needed. Of course, these results might be different for other genera that exhibit less homology among species and strains. Since *Brucella* is shed in milk in very low numbers, strain prediction remains a challenge that can be successfully tackled by reducing matrix background through the depletion of eukaryotic cells, as demonstrated in our study.

Metagenomic sequencing of sample number 151 resulted in >100 000 *Brucella *-specific reads after eukaryotic cell depletion. In this way, we could define the closest species with a similarity of >99 % using RefSNPer. Strain prediction with RefSNPer was even feasible with fewer reads (*n*=2787) without preceding eukaryotic cell depletion, in contrast to Mash Screen, which actually predicted the wrong species. We did not find any indications of a second *Brucella* strain in the milk sample after bioinformatics analysis with ConFindr [45] and sigma [46] (data not shown). Since RefSNPer might be unsuitable for distinguishing strains of the same species or genus in a mixed sample, we propose read extraction, for instance, with sparse and sigma as recently reported [47], before applying RefSNPer. We also tested two different assembly strategies for metagenomic sequence reads. *Brucella *-specific read extraction after Kraken2 classification, as previously described [5], and assembly with SPAdes yielded fewer contigs and a higher recovery of the genome fraction than metagenomic assembly by megahit. In a cgMLST analysis, the assembly after genus-specific read extraction (pre-assembly binning method) clustered closer to the isolates from the same milk sample than the metagenomic assembly of complete WMS data. When the megahit assembler was used for all data, the isolates clustered together with the metagenomic assembly and with the assembly of *Brucella *-specific reads. The dependence of cgMLST results on the assembler has been described previously [48]. The cgMLST analysis, which was performed with all *B. melitensis* assemblies available from the NCBI at that time, revealed four closely related strains, of which three also originated from Egypt. Since no further metadata of samples and strains were available, an epidemiological context could not be clarified. In the cgMLST analysis, the isolates in our study were more closely related to four publicly available genomes than to the WMS data obtained from the same milk sample. In contrast, SNP analysis revealed a closer association between isolates and WMS data, and is, therefore, more robust when WMS datasets shall be included. The metagenome was located in the same clade as the isolates, although WGS and WMS data differed in 11 nucleotide positions. Nevertheless, isolate sequences and WMS data can be clearly matched due to the majority of overlapping SNPs. SNP differences between isolates and metagenome might either be a consequence of insufficient sequence coverage at respective positions or due to multiple alleles in the sample. The latter is supported by the fact that the three isolates from the same milk sample differed at three nucleotide positions when directly compared to each other. Briefly, the striking resemblance of genomic and metagenomic data indicates the applicability of WMS in outbreak investigations.

In consistency with Lim and colleagues [13], we noticed differences in the composition of the milk microbiome depending on the DNA extraction method applied. These differences, however, were not statistically significant. Nevertheless, some families such as *Staphylococcaceae* and *Moraxellaceae* were underrepresented after eukaryotic cell depletion. This phenomenon was also observed in the Egyptian raw milk samples. Since *Staphylococcaceae* are Gram-positive bacteria and depletion of eukaryotic cells is in fact based on the selective lysis of mammalian cells due to specific membrane properties, we doubt that bacterial cells were lysed together with mammalian cells. In addition, insufficient lysis of Gram-positive bacteria can probably be ruled out by the slightly improved detection of *Corynebacteriaceae* after eukaryotic cell depletion. It is conceivable that the detection of *Staphylococcaceae* by metagenomics after conventional DNA extraction was based on the detection of soluble DNA, which was not detected when eukaryotic cell depletion was applied, because the selective lysis step also eliminates extracellular DNA. The presence of extracellular DNA is well known for members of the family *Staphylococcaceae* during biofilm formation. In addition, the depletion of extracellular DNA that distorts the actual microbial composition might be a desired effect if the aim is for only intact bacteria to be detected. The absence of several low-abundance families in naturally contaminated raw milk samples after eukaryotic cell depletion might also be a consequence of the methodological loss of extracellular DNA. By analysing negative controls (data not shown), we noticed that most of these differences in the abundance of *Bradyrhizobiaceae *, *Burkholderiaceae *, *Rhizobiaceae *, *Corynebacteriaceae* and *Pseudomonadaceae* originated from DNA contaminations within extraction kits known as the kitome [49].

Various AMR genes could be identified in the raw milk samples. Most of these genes showed a coverage of <90 %, which might emerge from low abundance, from partial similarities to other genes or from truncation. We observed more partial genes after conventional DNA extraction. Interestingly, there were also some high-certainty AMR genes (*n*=9), which were detected only after conventional DNA extraction. As mentioned above, this might be a consequence of the depletion of extracellular DNA along with eukaryotic DNA.

Source attribution studies applying metagenomics in foodstuffs are still rare. Most of these studies reported problems with the detection of low-abundance pathogens due to a high matrix background. While enrichment culture takes time and is not always a good alternative for fastidious pathogens, we could show that selective lysis of mammalian cells improved the detection of various pathogens in the complex milk matrix, including *Brucella* spp., *Salmonella enterica* and *E. coli *. In addition, our study has provided the proof-of-concept that metagenomics is a highly sensitive tool for microbial risk assessment of food, particularly when pathogens are difficult to isolate. In this way, we provide a simple approach for the detection and characterization of pathogenic bacteria in milk without pre-enrichment. Since purchasable kits and open-source software were used, our tool can be easily applied to other scientific questions and also to routine food microbiology.

## Supplementary Data

Supplementary material 1

Supplementary material 2
