# Supplementary material for: Direct identification and molecular characterization of zoonotic hazards in raw milk by metagenomics using Brucella as a model pathogen
Source: Microb Genom. 2021 May 4;7(5):000552. doi: 10.1099/mgen.0.000552 (PMC8209726; doi:10.1099/mgen.0.000552)
Supplement: Supplementary material 1 [file mgen-7-0552-s001.pdf]

Supplementary Figures

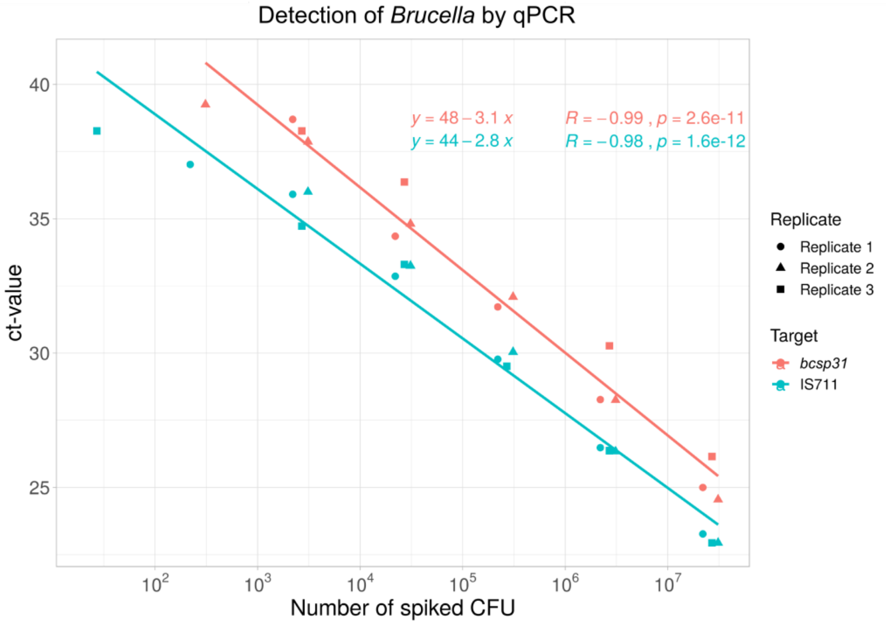

Figure S1: Detection of *Brucella* by qPCR in artificially inoculated raw milk. Ct-values of three biological replicates are shown with increasing concentration of bacterial cells using two different genus-specific targets, namely *bcsp31* (red) and IS711 (blue). Regression lines and their formula, as well as correlation coefficients (R) with p-values (p) are displayed.

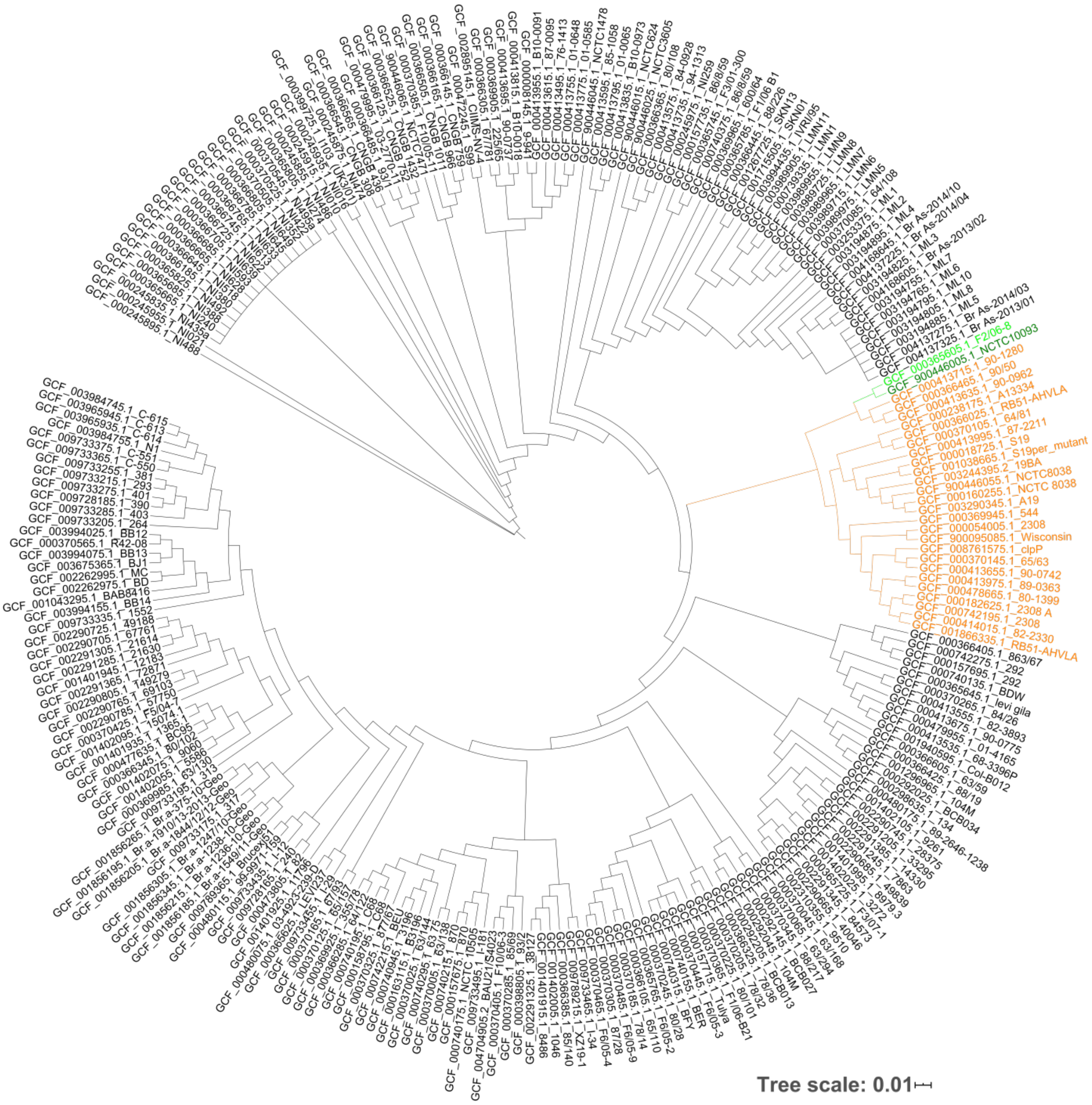

Figure S2: Clustering of *Brucella abortus* strains in a maximum-likelihood tree based on core genome SNPs. A core genome SNP-analysis was performed with 249 publicly available draft and complete *B. abortus* genomes retrieved from NCBI. To ease presentation the phylogenetic branch lengths were ignored. The clade and subclade containing *Brucella abortus* strain 544 (NCTC10093) were highlighted in orange and green, respectively.

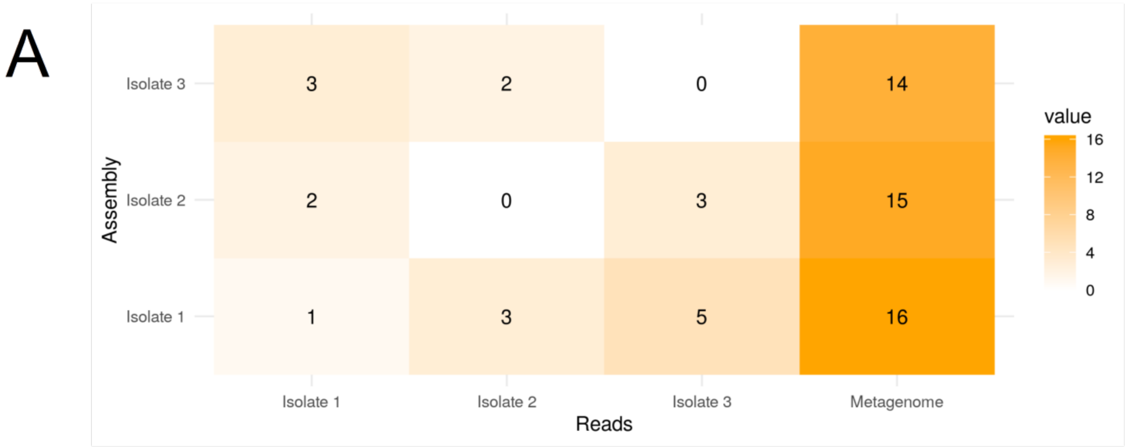

Figure S3: SNP-matrix of pairwise comparison of assembled sequencing reads derived from WGS of isolates and WMS of raw milk (sample no. 151). The number of SNPs after mapping reads to assemblies from WGS data of isolates was determined. Only SNPs covered by ≥3 reads were counted.

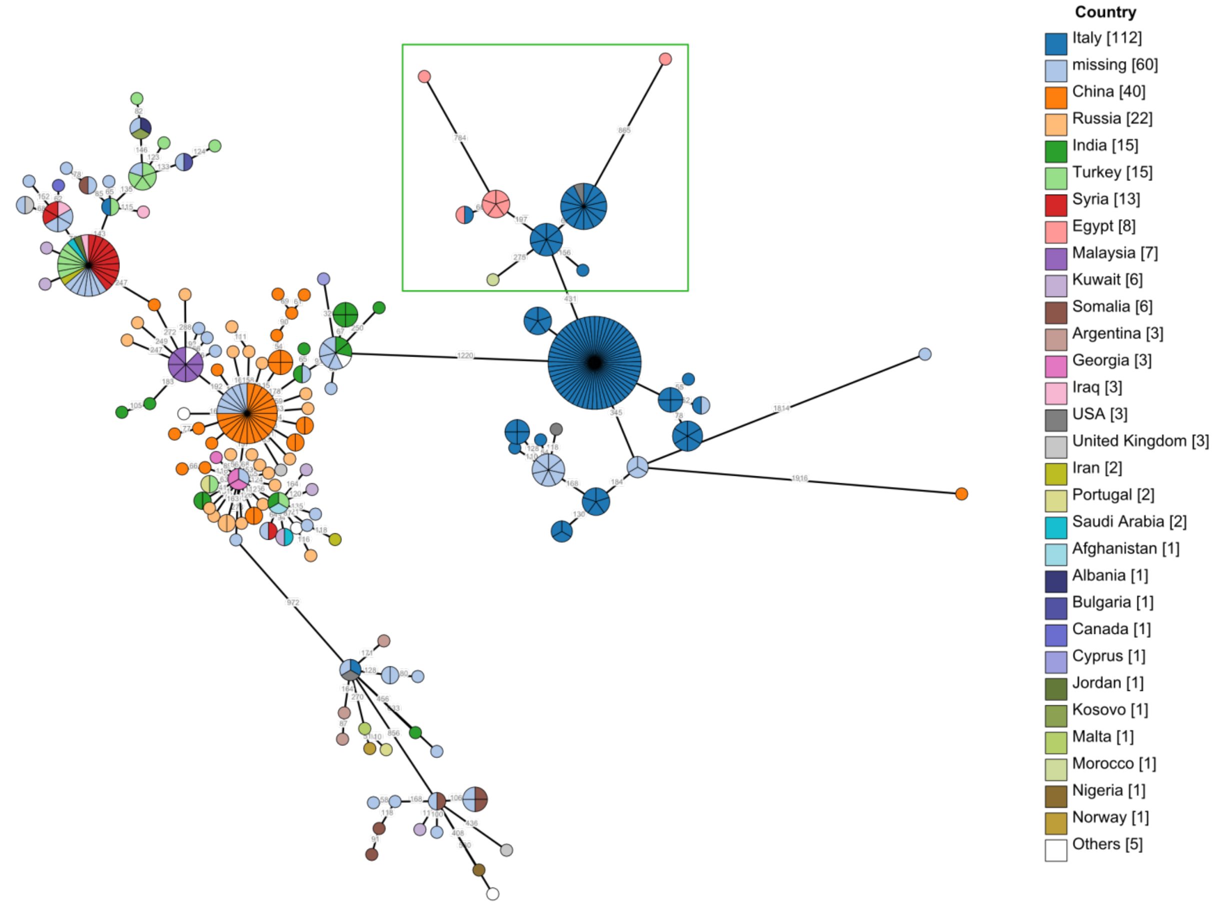

Figure S4: Clustering of *Brucella melitensis* strains in a minimum spanning tree based on cgMLST allelic profiles. The cgMLST analysis was performed on assemblies of isolates and metagenomic datasets from raw milk sample no. 151 and 336 publicly available draft and complete *B. melitensis* genomes retrieved from NCBI. The country of origin of the strains is color coded. Allelic distances are

displayed as figures at the branches, and branches revealing less than 50 alleles difference are collapsed. The picture section framed in green is shown in more detail in Figure 4.
